# Cognitive impairment due to ischemic stroke in female rats is associated with secondary neurodegeneration, and attenuated with microRNA‐20a‐3p as assessed by diffusion tensor MR imaging and histological analysis

**DOI:** 10.1002/alz.094158

**Published:** 2025-01-09

**Authors:** Dayalan Sampath, Jacob Singer, Balaji Gopalakrishnan, Zara Akbari, Monserrat Villarreal, David A Hurst, Brenda P Noarbe, Andre Obenaus, Farida Sohrabji

**Affiliations:** ^1^ Texas A&M University Health, Bryan, TX USA; ^2^ University of California Irvine, Irvine, CA USA

## Abstract

**Background:**

Our studies show that the small non‐coding RNA, mir20a‐3p, is neuroprotective for stroke in the acute phase and also attenuates long term cognitive decline in middle‐aged female rats. Cognitive decline due to vascular diseases, such as stroke, is associated with secondary neurodegeneration in cortex and limbic structures. In this study, we assessed the volume of white matter, ventricles and regional diffusion‐weighted MR imaging measures to delineate pathological tissue characteristics from the postmortem brain of stroke rats.

**Method:**

Middle‐aged females were subjected to ischemic stroke using the vasoconstrictor endothelin 1, injected adjacent to the left middle cerebral artery (MCA). Mir‐20a‐3p mimic (n=10) or scrambled oligo (n=8) was administered i.v. 4h, 24h and 70d post stroke. Animals were assessed periodically for cognitive performance up to 100d after stroke using both the cued fear conditioning test and the novel object recognition test (NORT). After perfusion fixation, T2 and diffusion tensor imaging (DTI) magnetic resonance imaging were performed on postmortem tissue. Subsequently, brains were processed for histology, including the Weil myelin stain.

**Result:**

Stroke resulted in impairment in both the cued fear conditioning test and the NORT, which was attenuated by mir‐20a‐3p treatment (Sampath et al., 2023). Weil myelin‐stained sections showed that both the corpus callosum and internal capsule had a reduction in volume in the ischemic hemisphere as compared to non‐ischemic hemisphere in vehicle treated (scrambled oligo) MCAo animals. In contrast, sham and MCAo+Mir‐20a‐3p animals displayed no hemispheric differences in the volume in either tract. A similar volume asymmetry was also observed in the ventricles in the MCA0+ Scrambled group. Entorhinal cortex volume (diffusion tensor MR images) was also asymmetric in the MCAo+scrambled oligo group.

**Conclusion:**

Cognitive changes measured with remote fear memory retrieval and NORT were associated with reduced density of major forebrain white matter tracts due to stroke and enlarged ventricular volume indicative of cell loss. This was observed in the entorhinal cortex, a region involved with contextual memory processing. These stroke‐induced deficits were attenuated in Mir‐20‐3p treated animals, indicating that microRNA treatment attenuated secondary neurodegeneration.

